# Combined Effects of Intermittent Hypoxia and Amyloid Beta on Hippocampal Activity, Its Cholinergic Modulation, and Memory

**DOI:** 10.1002/hipo.70017

**Published:** 2025-06-11

**Authors:** Pinedo‐Vargas Laura, Méndez‐Salcido Felipe, Lorea‐Hernández Jonathan‐Julio, Victor de Lafuente, Peña‐Ortega Fernando

**Affiliations:** ^1^ Departamento de Neurobiología del Desarrollo y Neurofisiología Instituto de Neurobiología, Universidad Nacional Autónoma de México Querétaro México

**Keywords:** acetylcholine, Alzheimer disease, memory, neural network activity, sleep apnea, spike activity

## Abstract

Obstructive sleep apnea (OSA), characterized by repetitive upper airway obstruction, leads to chronic intermittent hypoxia (cIH) and induces cognitive and neuronal network disruptions similar to those observed in Alzheimer's disease (AD). These pathologies are often presented together in the elderly and share some pathophysiological mechanisms. The presence of amyloid beta (Aβ), observed both in AD and OSA patients, can alter brain function, cholinergic modulation and memory either independently or in addition with cIH. To explore these possibilities, we studied the pathological effects of Aβ, cIH, and their combination on cognition and hippocampal activity, and its modulations by the cholinomimetics carbachol, muscarine, and nicotine, along with evaluations of choline acetyltransferase (ChAT) expression in the septum and the hippocampus. We found that cIH and Aβ similarly affect spatial memory and additively impact aversive memory (sparing recognition memory). Although cIH and Aβ share some pathological effects on hippocampal activity and its modulation by cholinomimetics, when combined, they produce an additive inhibition at the population and single‐cell levels more evident in the presence of nicotine. No change in ChAT expression was observed. Remarkably, the departure from normal firing as well as the disruption of carbachol‐induced response correlates with the reduction of aversive memory in our experimental groups. In summary, cIH and Aβ share pathological effects, but their combination exacerbates functional pathology, contributing to our understanding of AD/OSA pathophysiology and co‐morbidity.

## 1 Introduction

Obstructive sleep apnea (OSA) is characterized by recurring upper airway interference resulting in episodic apneas and intermittent hypoxemia (Bubu et al. 2020; Osorio et al. 2015). Thus, OSA can be experimentally simulated by chronic intermittent hypoxia (cIH, Arias‐Cavieres et al. 2020; Hernández‐Soto et al. 2021). OSA or cIH lead to cognitive impairments, to alterations in hippocampal function (Bubu et al. 2020; Osorio et al. 2015; Arias‐Cavieres et al. 2020), and its modulation by acetylcholine (Row et al. 2007), similar to those observed in Alzheimer's disease (AD; Gutiérrez‐Lerma et al. 2013; Owen et al. 2021).

OSA and AD share pathophysiological mechanisms and may have an interdependent relationship regarding their progression (Bubu et al. 2020; Osorio et al. 2015; Liguori et al. 2017). For instance, OSA patients show a higher rate and an earlier emergence of AD (Bubu et al. 2020; Osorio et al. 2015), while treating OSA delays AD onset (Osorio et al. 2015), improves cognition (Liguori et al. 2017) and reduces AD‐neuropathology (Owen et al. 2021). On the other hand, patients with AD have a higher risk of OSA than cognitively intact individuals of similar age (Emamian et al. 2016; Gaeta et al. 2020).

Both AD and OSA are associated with amyloid beta (Aβ) accumulation (Bubu et al. 2020; Ju et al. 2019; Owen et al. 2021; Kong et al. 2021), which depends on OSA severity (Owen et al. 2021), correlates with cognitive impairment (Kong et al. 2021) and is reduced with OSA treatment (Ju et al. 2019). Considering that OSA patients showing high Aβ levels are more likely to progress to AD (Bubu et al. 2020), it is likely that cIH and Aβ exert additive pathological actions (Bubu et al. 2020). Additionally, AD and OSA affect the cholinergic system (Gutiérrez‐Lerma et al. 2013; Nardone et al. 2016), which strongly modulates neuronal network function and cognition and is a major target for OSA and AD treatment (Hambrecht et al. 2007; Row et al. 2007; Sotthibundhu et al. 2008; Knowles et al. 2009; Gutiérrez‐Lerma et al. 2013; Dannenberg et al. 2017; Marra et al. 2019; Shen et al. 2021). Acetylcholine exerts a complex and state‐dependent modulation of hippocampal network activity (Bell et al. 2013; Wang et al. 2015; Yang et al. 2020), mainly due to diverse responses elicited by the activation of their various nicotinic (de la Garza et al. 1987; Wong and Gallagher 1989, 1991; Mansvelder et al. 2006; Wang et al. 2015; Dannenberg et al. 2017; Bueno‐Junior et al. 2017) and muscarinic receptors (McQuiston and Madison 1999; Widmer et al. 2006; Dannenberg et al. 2017; Yang et al. 2020), as well as their differential effects on pyramidal cells and interneurons (McQuiston and Madison 1999; Widmer et al. 2006; Dannenberg et al. 2017; Yang et al. 2020); all of which depend on the overall network excitability and/or its neuromodulatory status (Somogyi and de Groat 1992; Bell et al. 2013; Plata et al. 2013; Wang et al. 2015; Dannenberg et al. 2017; Bueno‐Junior et al. 2017; Yang et al. 2020). Remarkably, a comparative study of the pathological actions of cIH, Aβ, and their combination on cholinergic modulation and markers is missing.

AD and OSA patients also exhibit EEG “slowing” (i.e., reduction in high frequency activity and an increase in slower ones; Muñoz‐Torres et al. 2020), that can be partially corrected with OSA treatment (Lee et al. 2012; Ju et al. 2019), that is related to OSA severity (Appleton et al. 2019; Kang et al. 2021; Muñoz‐Torres et al. 2020), and is reproduced by Aβ (Gutiérrez‐Lerma et al. 2013; Peña‐Ortega 2013, 2019), further indicating that cIH and Aβ may similarly affect network activity (Gutiérrez‐Lerma et al. 2013; Hernández‐Soto et al. 2019, 2021; Peña‐Ortega 2013, 2019), its cholinergic modulation (Row et al. 2007; Gutiérrez‐Lerma et al. 2013), and cognition (Salgado‐Puga et al. 2017; Arias‐Cavieres et al. 2020).

Despite the similarities described in the previous paragraphs and the high AD/OSA co‐morbidity rate, a comparative study of the pathological actions of cIH, Aβ, and their combination on memory, neural activity, and its modulation by acetylcholine is missing. Thus, we evaluated the effects of cIH, Aβ, and cIH+Aβ on hippocampal‐dependent cognition, hippocampal population and single‐cell activity, their response to the cholinomimetics carbachol (Cch; Gutiérrez‐Lerma et al. 2013), muscarine, and nicotine (Bell et al. 2013; Wang et al. 2015; Yang et al. 2020), and the expression of the cholinergic marker choline acetyltransferase (ChAT) in the septum and the hippocampus. We found that cIH and Aβ produce some convergent but also divergent effects on hippocampal‐dependent memory, on population and single‐cell hippocampal activity, as well as on their response to cholinomimetics, without any change in ChAT expression. We also found that the combination of cIH and Aβ adds to their individual pathological actions in memory avoidance and hippocampal activity, providing cellular foundations for the morbid influences between AD and OSA.

## 2 Methods and Materials

### 2.1 Animals

Experiments were approved by the Bioethics Committee of the Institute of Neurobiology (INb)‐UNAM and were performed in accordance with the Official Mexican Norm for the Use and Care of Laboratory Animals (*Norma Oficial Mexicana* NOM‐062‐ZOO‐1999). C57BL/6 mice (*n* = 70; 8 weeks old) were obtained from the INb‐UNAM Animal Facility, housed in collective cages (3–4 animals/cage) and maintained in a vivarium with controlled temperature (23°C ± 1°C), under a normal light–dark cycle (12/12 h; lights on at 7:00 a.m.). Animals were supplied with food (LabDiet 5001) and water *ad libitum* before the experiments. Transgenic mice of the following strains: 3xTg‐AD (B6;129‐Tg(APPSwe, tauP301L)1Lfa Psen^1tm1Mpm^/Mmjax; The Jackson Laboratory) and 5xTg‐AD (B6.Cg‐Tg (APPSwFlLon, PSEN1*M146L*L286V)6799Vas/Mmjax; The Jackson Laboratory) were kindly provided by Dr. Sofía Díaz‐Cintra, INb‐UNAM, and housed in the same conditions.

### 2.2 Aβ Administration

Mice were intracerebroventricularly microinjected with 5 μL Aβ (100 pmoles/μL) or its vehicle (F12 medium). To do so, animals were anesthetized with sevoflurane 2% and maintained with sevoflurane 0.6% (Peña and Tapia 1999, 2000). Aβ_1–42_ was oligomerized using a previously described protocol (Balleza‐Tapia et al. 2010; Cornejo‐Montes‐de‐Oca et al. 2018; Hernández‐Soto et al. 2019), in which Aβ is finally dissolved in F12 medium, making it the vehicle for the experiments (Balleza‐Tapia et al. 2010; Cornejo‐Montes‐de‐Oca et al. 2018; Hernández‐Soto et al. 2019; Martínez‐García et al. 2021). This oligomerized Aβ_1–42_ solution contains different forms of oligomers, as well as monomers and protofibrils (Balleza‐Tapia et al. 2010; Cornejo‐Montes‐de‐Oca et al. 2018; Hernández‐Soto et al. 2019; Martínez‐García et al. 2021). After verifying proper anesthesia levels by the absence of pedal reflexes, animals were affixed to a stereotaxic frame. Then, a small craniotomy was performed for the microinjection, which was achieved with a stainless‐steel needle connected to a Hamilton syringe (10 μL; Hamilton Company) via plastic tubing and controlled by a microinfusion pump (World Precision Instruments). The following coordinates were used for the intracerebroventricular infusion: AP −0.9 mm, ML + 1.7 mm, and DV −2.2, with respect to Bregma and skull surface (Franklin and Paxinos 2001). At this location, either 5 μL of Aβ (100 pmoles/μL) or vehicle was microinjected at a rate of 0.5 μL/min. The microinjector was left in place for 10 min to allow proper solution diffusion. A dose of up to Aβ 500 pmoles, but not less, is required to induce cognitive impairment in rats (Salgado‐Puga et al. 2015) and mice (Raj et al. 2019). This Aβ dose does not induce cell damage in the olfactory bulb, the prefrontal cortex, or the hippocampus (Alvarado‐Martínez et al. 2013; Salgado‐Puga et al. 2015; Torres‐Flores and Peña‐Ortega 2022). To reach this dose, 5 μL of a 100 μM Aβ solution is required (Salgado‐Puga et al. 2015). We confirmed that the infusion of such volume of vehicle does not induce microgliosis or major changes in hippocampal microglial morphology 3 weeks later (Figure S1). In fact, we only found that hippocampal microglia in the injected hemisphere increases its area and perimeter compared to microglia from the contralateral hemisphere (by 27% and 37%, respectively) or to microglia from sham animals (by 19% and 17%, respectively; Figure S1). The circularity and solidity of hippocampal microglia in the injected hemisphere decreased only when compared to its contralateral hemisphere (by −34% and −18%, respectively; Figure S1), but were indistinguishable from microglial from sham animals (Figure S1). All other morphometric parameters or microglial density remain unchanged, indicating that this amount of fluid does not induce major brain inflammation. By evaluating microglial morphology 3 weeks after the microinjections, we might be unable to capture the immediate response following injection but aimed to reveal the microglial state while animals underwent the behavioral and electrophysiological evaluations later described.

### 2.3 Chronic Intermittent Hypoxia

One week after surgery, cIH was induced as previously reported (Villasana‐Salazar et al. 2020; Hernández‐Soto et al. 2021; Camacho‐Hernández et al. 2022). Briefly, every day, mice were placed in collective cages (3–4 animals/cage) within Plexiglas chambers (76 cm × 51 cm × 51 cm) equipped with gas injectors and with O_2_, CO_2_, humidity, and temperature sensors (BioSpherix, NY, USA). With an OxyCycler system (A42OC, BioSpherix), animals were submitted to intermittent substitution of O_2_ in the chambers for N_2_ for 5 min to induce hypoxic episodes (5%–6% inspired O_2_) followed by replenishment of O_2_ for 1 min during normoxic episodes (21%–22% inspired O_2_; Villasana‐Salazar et al. 2020; Hernández‐Soto et al. 2021; Camacho‐Hernández et al. 2022), which were cyclically alternated for 8 h daily (from 10:00 to 18:00 h) during the light phase of the cycle for 14 days (Villasana‐Salazar et al. 2020; Hernández‐Soto et al. 2021; Camacho‐Hernández et al. 2022); control animals underwent the same protocol in constant normoxic conditions (21% O_2_; Villasana‐Salazar et al. 2020; Hernández‐Soto et al. 2021; Camacho‐Hernández et al. 2022).

### 2.4 Behavioral Tests

Behavioral tests were performed 2 days apart from each other at the Institute of Neurobiology‐UNAM Behavioral Analysis Facility in the following order (to minimize the confounding effects of a stressful task): novel object recognition (NOR) or novel object location (NL) were counterbalanced as the first test (followed by the other one). Then, inhibitory avoidance was performed (Zarrindast et al. 2002; Salgado‐Puga et al. 2017). Inhibitory avoidance was evaluated only once and always as the last behavioral test to avoid any interference due to stress induced by the electrical shock. In contrast, NOR and NL tests were performed before any other experimental manipulation and repeated at the end of the experimental manipulations just before the inhibitory avoidance. Animals that did not show a proper performance at the initial NOR and NL evaluations were excluded from the study (15% of tested animals; Xolalpa‐Cueva et al. 2022). Tests were performed after 5 days of habituation to the testing room, the testing arena as well as to the experimenter. Namely, animals were gently handled for 3 days (Two 5‐min‐long sessions per day). Then, animals were habituated to the arena for 10 min for two consecutive days before further testing.

#### 2.4.1 Novel Object Recognition (NOR) Test

For this test, we used a black circular arena, to which animals were previously familiarized where two identical objects (6.3 cm plexiglass cubes) were located for the animal to explore freely for 10 min (Xolalpa‐Cueva et al. 2022). Then, the animals returned to their home cage. To avoid olfactory cues, sawdust was replaced, and the test arena and the objects were thoroughly cleaned with ozonized water after each trial. Twenty‐four hours after familiarization with the original objects, animals were returned to the testing arena, which conserved one of the original objects (in its original position) and also contained a new object (a 7.0 high and 6 cm long plexiglass object with an hourglass shape; located in the position of the removed original object). The animals were left in these conditions for 5 min while their exploration was evaluated (Xolalpa‐Cueva et al. 2022).

#### 2.4.2 Novel Object Location (NL) Test

For this test we used the same circular arena, where two identical objects (6.0 cm high and 6.7 cm tall glass cylindrical jars with metallic cape; different to those used in NOR) were located for the animal to explore them freely for 5 min. Then, the animal returned to its home cage. Sawdust was replaced, and the test arena and the objects were thoroughly cleaned with ozonized water after each trial. Twenty‐four hours after the familiarization with the original objects and locations, animal was returned to the testing arena which conserved one of the original objects in its original position and the other object was displaced 10 cm to a novel location. The animal was left in these conditions for 5 min while its exploration was evaluated (Xolalpa‐Cueva et al. 2022).

All sessions were videotaped with a camera placed over the arena. The analysis of the time spent exploring the two objects was made offline. Exploration was considered when the animal pointed the nose to an object at 2 cm or less and/or touched it with the nose. The preference index (*P*_idx_) was calculated as the percentage of the total exploratory time spent at each object or location, as appropiate (Xolalpa‐Cueva et al. 2022).

#### 2.4.3 Inhibitory Avoidance

This single‐trial, step‐through, inhibitory avoidance task was only performed once at the end of all experimental manipulations, as previously described (Salgado‐Puga et al. 2015, 2017). Briefly, the training apparatus was divided into two compartments (one illuminated and one dark) separated by a guillotine door. The safe compartment has a floor of stainless‐steel bars and a light bulb located in the center of its lid. The dark V‐shaped shock compartment has walls and a floor made of stainless‐steel plates. A slot separates the two stainless steel plates that were electrified using a square‐pulse stimulator (Grass S‐48) in series with a constant current unit (Grass CCU‐1A). The training apparatus was located inside a dark, soundproof room provided with background masking noise. For training, the animal was placed in the safe compartment and 10 s later, the guillotine door was opened, and the latency to enter the shock compartment was recorded (training latency). Once the animal was completely inside the dark chamber, the door was closed, and a 4 s‐long foot‐shock (0.5 mA) was delivered. After 5 s, the door was opened, allowing the animal to escape into the safe compartment. Once the animal was in the safe compartment, the door was closed; the animal was left there for 10 s and then put back in its home cage. Memory evaluation (retention test) was performed 24 h later, and the latency to enter the shock compartment was measured (memory latency). The test ended either when the animal entered the dark compartment or after 180 s without entry, in which case a score of 180 was assigned. If the animal entered the dark compartment during the retention test, the foot‐shock was not delivered.

### 2.5 Electrophysiology

#### 2.5.1 Slice Preparation

After behavioral evaluations, horizontal slices were obtained from the dorsal hippocampus (Adaya‐Villanueva et al. 2010; Gutiérrez‐Lerma et al. 2013). To do so, animals were anesthetized with sodium pentobarbital (70 mg/kg) and perfused transcardially with cold protective saline containing 238 mM sucrose, 3 mM KCl, 2.5 mM MgCl_2_, 25 mM NaHCO_3_, and 30 mM d‐glucose, pH 7.4, and bubbled with carbogen (95% O_2_ and 5% CO_2_). Then, animals were decapitated, and their brains were removed and dissected in ice‐cold artificial cerebrospinal fluid (aCSF) containing 119 mM NaCl, 3 mM KCl, 1.5 mM CaCl_2_, 1 mM MgCl_2_, 25 mM NaHCO_3_, and 30 mM d‐glucose, pH 7.4, and bubbled with carbogen (95% O_2_ and 5% CO_2_). Horizontal 400 μm thick slices, containing the dorsal hippocampal formation, were cut with a vibratome (HM‐650 V Thermo Scientific). Slices were left to recover in aCSF at room temperature for at least 60 min before any further experimental manipulation.

#### 2.5.2 Multielectrode Arrays (MEA) Recordings

After recovery, a horizontal slice was placed on a 60‐electrode multielectrode array (MEA, 6 × 10 grid, 60pMEA100/30iR‐Ti, Multichannel Systems; Méndez‐Salcido et al. 2022) perfused above and below with circulating aCSF at 37°C and bubbled with carbogen. The electrodes covered most of the CA1 region and the subiculum. Negative pressure was applied, transiently, to the bottom perfusion system to secure the slice's attachment to the electrodes (Nieto‐Posadas et al. 2014; Juárez‐Vidales et al. 2021; Méndez‐Salcido et al. 2022). Electrophysiological signals were left to stabilize for 30 min. The raw extracellular signal was recorded at 25 kHz using the MEA2100 system and MC‐Rack software (Multichannel Systems). In one set of experiments, after baseline recordings, Cch was bath applied at 5 μM and later raised to 10 μM (Gutiérrez‐Lerma et al. 2013). In another set of experiments, muscarine 2.5 μM was bath applied and later followed by nicotine 100 μM (in the continuous presence of muscarine). In each case, 15 min of recordings were obtained just 3 min after the beginning of each condition. MEA recordings were preprocessed and, subsequently, local field potentials (LFP; Adaya‐Villanueva et al. 2010; Gutiérrez‐Lerma et al. 2013) and firing activity (Nieto‐Posadas et al. 2014; Juárez‐Vidales et al. 2021; Méndez‐Salcido et al. 2022) were analyzed further.

For the LFP analysis, the signal was downsampled to 1 kHz and low‐pass filtered below at 350 Hz using MC_Rack software (Multichannel Systems). The power spectral density of the signal was obtained with the pWelch function from Matlab (Version 2020b) for frequencies below 200 Hz. For the recordings in the presence of Cch or muscarine, the power was normalized by setting as 100% the mean power of all channels during basal conditions, and the normalized power of all recordings was calculated as % of basal (Adaya‐Villanueva et al. 2010). For the recordings after the application of nicotine, in the continuous presence of muscarine, the power was normalized by setting as 100% the mean power of all channels during muscarine‐only presence, and the normalized power of all recordings was calculated as % of muscarine. The power spectra were segmented in bands of interest as follows: Delta/Theta (*δ*/*θ*: 1–15 Hz), Beta (*β*: 16–28 Hz), slow‐Gamma (*sγ*: 29–45 Hz) and fast‐Gamma (*fγ*: 75–200 Hz). For spiking activity, the signal was band‐pass filtered at 350–7000 Hz using MC_Rack. Channels containing clear neural spikes were exported to the Offline Sorter software (Plexon), and spikes were identified by thresholding (4.0 standard deviations [SD] of the whole signal). Spike sorting was carried out by performing principal component analysis over the identified spike waveforms (Nieto‐Posadas et al. 2014; Juárez‐Vidales et al. 2021; Méndez‐Salcido et al. 2022), followed by clustering using a supervised Valley‐Seeking 3D algorithm.

### 2.6 Histology and Immunofluorescence

For the immunohistochemical detection of ChAT, Aβ or Iba‐1 (Méndez‐Salcido et al. 2022; Torres‐Flores and Peña‐Ortega 2022; Salas‐Gallardo et al. 2024), as well as for tissue staining with Thioflavin S and Thiazine Red (Alvarado‐Martínez et al. 2013), whole brains were obtained from an independent set of animals from all experimental groups. Animals were anesthetized with an intraperitoneal injection of sodium pentobarbital (70 mg/kg) and perfused transcardially with a cold saline solution containing 238 mM sucrose, 3 mM KCl, 2.5 mM MgCl_2_, 25 mM NaHCO_3_, and 30 mM D‐glucose, pH 7.4 and bubbled with carbogen (95% O_2_ and 5% CO_2_). Then, animals were decapitated, and their brains were dissected and fixed in 4% paraformaldehyde (PFA) at pH 7.4 for 24 h. After fixation, brains were immersed in phosphate‐buffered saline (PBS) containing 30% sucrose until sinking. Subsequently, 30 μm‐thick sections were obtained with a cryostat (Leica Biosystems, Wetzlar, Germany). Free floating sections were processed under constant agitation.

A set of tissue sections was incubated with one of the following antibodies at the dilutions suggested by manufacturers and validated in previous publications (including our own): anti‐Amyloid β42 (Thermo Fisher #44‐344, at 1:500 dilution; Zhang et al. 2014; Rostagno et al. 2022), anti‐ChAT (Sigma‐Aldrich #HPA048547, at 1:800 dilution; Hatton et al. 2020; Xie et al. 2024) or anti‐Iba1 (Wako Chemicals #019‐19741, at 1:1000 dilution; Fernández‐Arjona et al. 2017; Green et al. 2022; Méndez‐Salcido et al. 2022) in PBS containing 0.5% Triton X‐100 for 48 h at 4°C. Then, sections were washed with PBS and subsequently incubated with either one of the following secondary antibodies: Alexa Fluor 568 or Alexa Fluor 488 anti‐Rabbit (Thermo Fisher, at 1:500 dilution) in PBS containing 0.5% Triton X‐100 at room temperature for 2 h. Then, sections were washed with PBS and incubated with DAPI (Thermo Fisher, at 1:1000 dilution) in PBS for 1 min. After this, washed sections were mounted on glass slides, embedded in Entellan (Merck) and covered with glass coverslips.

Other set of tissue sections was incubated with sodium borohydride (Sigma Aldrich, at 1% in PBS), washed with PBS, and sequentially incubated with the following dyes: Thioflavin S (Sigma Aldrich, at 0.005% in 80% ethanol) or Thiazine Red (Sigma Aldrich, at 0.0001% in deionized water) for 15 min (sections were washed with PBS in between and at the end). Washed sections were mounted on glass slides, embedded in Entellan (Merck) and covered with glass coverslips. Fluorescence micrographs were acquired using an Axio Imager 2 microscope (ZEISS) coupled to an Axiocam 305 camera (ZEISS).

### 2.7 Image Processing and Quantification

Micrographs were processed with the ImageJ‐based program Fiji. Brightness and contrast were automatically adjusted to improve visualization. The amount of ChAT‐positive septal neurons was counted in a circular area of 6 × 10^5^ μm^2^ located 200 μm rostral to the anterior commissure and 1 mm above the ventral edge, excluding the vertical nucleus of the diagonal band of Broca and striatum in up to 5 sections per animal, 3–4 animals per group (Yan et al. 2018). Only sagittal sections corresponding to the medial septal portion (up to 250 μm from the interhemispheric fissure) were included in quantification. All sections that did not allow the placement of the reference area were excluded. A neuron was considered ChAT‐positive when its fluorescence exceeded the mean background fluorescence value plus 2.7 times its standard deviation. The amount of microglia was counted in a circular area of 2.5 × 10^5^ μm^2^ located at the interface between the subiculum and the CA1 hippocampal regions. For the morphological characterization of microglia and for the quantification of ChAT‐positive axon/terminals in the hippocampus, images were converted to an 8‐bit grayscale format and binarized using MaxEntropy thresholding.

After the binarization of hippocampal Iba‐positive images, only clearly isolated microglia were included in the following analysis (up to 5 cells per section, 3 sections per animal, at least 3 animals per group), for which the following parameters were calculated: area (μm^2^), perimeter (μm), circularity (4 *π* area/perimeter^2^), aspect ratio major axis/minor axis, roundness: 4π* (area/π* major axis^2^), and solidity (area/convex area). These calculations were performed using the ‘Measure’ function in the ‘Analyze’ section of Fiji. These parameters are highly relevant for determining morphological differences and changes in microglial states (Fernández‐Arjona et al. 2017; Salas‐Gallardo et al. 2024).

After the binarization of hippocampal ChAT‐positive images, a circular area of 2 × 10^4^ μm^2^ was positioned at the interface between the subiculum and CA1 hippocampal regions (3 sections per animal, 3 at least animals per group) and the area covered by ChAT‐positive structures was quantified with the ‘Analyze Particles’ function, excluding particles ≤ 1 μm^2^. The percentage of area covered was calculated as follows: (ChAT structures area/reference area) * 100.

The fluorescence intensity was obtained, using “measure” function in Fiji, for all types of amyloid‐beta staining (3 sections per animal, 3 at least animals per group) in a circular area of 2 × 10^4^ μm^2^ placed at the interface between the subiculum and CA1 hippocampal regions, avoiding the inclusion of highly condensed structures, such as amyloid plaques in transgenic animals.

### 2.8 Statistical Analyses

**FIGURE 1:**
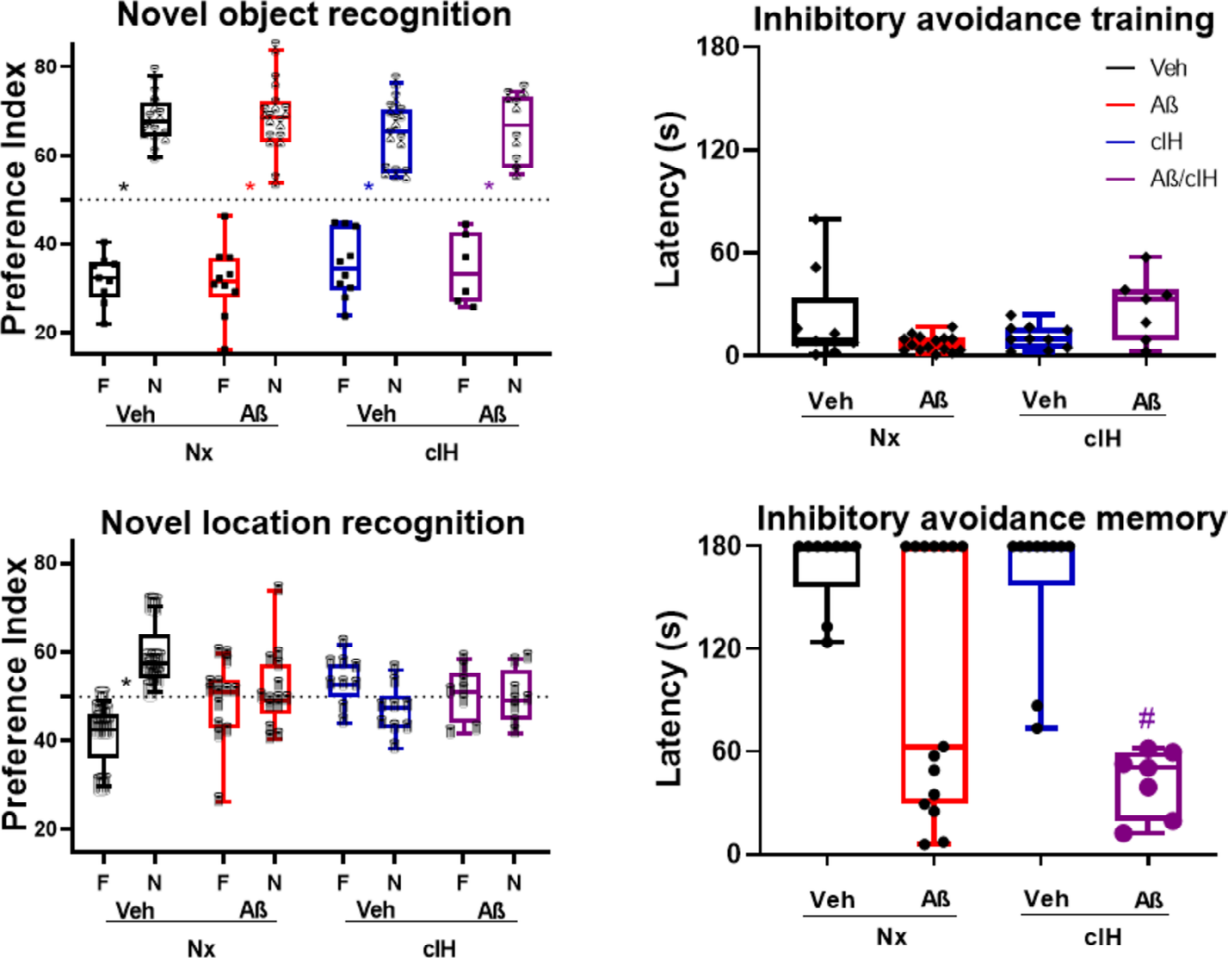
Memory evaluations. Left graphs: Preference indexes for familiar (F) and novel (N) objects for animals treated with vehicle (Veh) and with Aβ breathing normoxic air (Nx), as well as animals treated with chronic intermittent hypoxia (cIH) and its combination with Aβ (Aβ/cIH) in the Novel Object Recognition Test (NOR; *n* = 9,10,10 & 6; respectively) and the Object Location Recognition Test (NL; *n* = 9,14,9 & 8; respectively). The shape of the symbol depicting each data point resembles the actual novel object used in NOR and the identical objects used in NL. Right graphs: Latencies to enter the dark compartment for the groups Veh (*n* = 9), Aβ (*n* = 15), cIH (*n* = 10) and Aβ/cIH (*n* = 7) are shown for the training (top) and the memory phase (bottom) of the inhibitory avoidance test. # denotes *p* < 0.05 vs. Veh, also highlighted by bigger circles colored as the actual group; Wilcoxon rank‐sum test for independent samples; * denotes a *p* < 0.05 between F and N indexes, also highlighted by bigger symbols colored as the actual group in NL; Wilcoxon signed‐rank test for paired samples.

**FIGURE 2:**
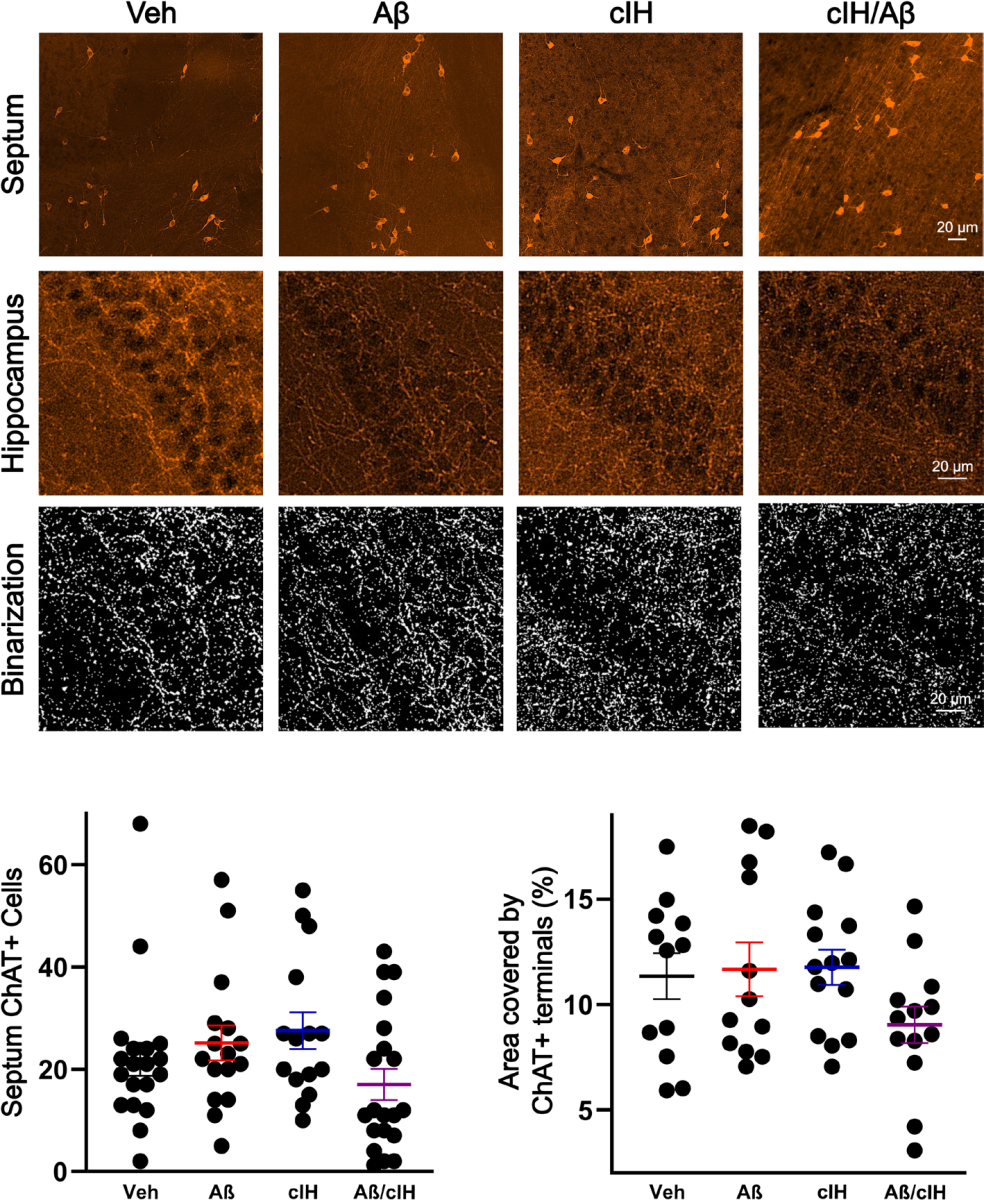
Septal cholinergic neurons and cholinergic hippocampal innervation. Representative micrographs of septal ChAT‐positive cells (upper row) and of ChAT‐positive axon/terminals in the hippocampus (middle row) are shown for all groups. Corresponding binarized images of the ChAT‐positive axon/terminals in the hippocampus are also shown (bottom row). Left graph: Quantification of ChAT‐positive cells in an area of 6 × 10^5^ μm^2^ located in the septum. The mean and standard errors are included with the following color code: Vehicle in black (*n* = 19 sections, 4 animals), Aβ in red (*n* = 16 sections, 4 animals), cIH in blue (*n* = 15 sections, 3 animals), and combination in purple, (*n* = 20 sections, 4 animals). Right graph: Quantification of ChAT‐positive axon/terminals in an area of 2 × 10^4^ μm^2^ located in at the interface between the subiculum and CA1 regions of the hippocampus for the following groups: Vehicle (mean and standard error in black, *n* = 12 sections, 4 animals), Aβ (mean and standard error in red, *n* = 12 sections, 4 animals), cIH (mean and standard error in blue, *n* = 14 sections, 3 animals) and combination (mean and standard error in purple, *n* = 13 sections, 4 animals).

**FIGURE 3:**
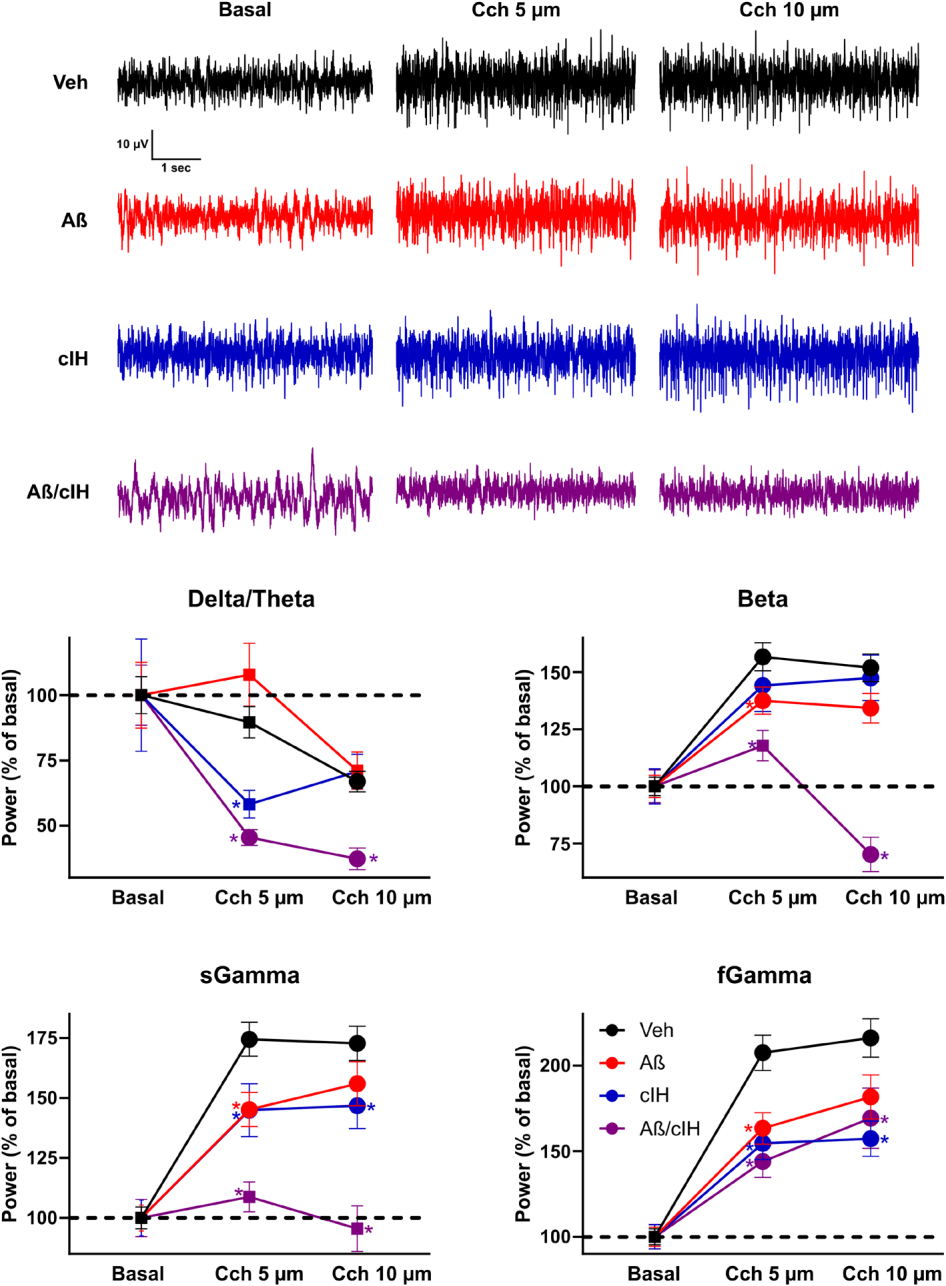
Modulation of hippocampal population activity by carbachol (Cch). Representative broadband traces of hippocampal recordings before (basal) and during the bath application of Cch 5 μM and 10 μM are shown for all the experimental groups. Normalized power (basal set as 100%) of the response to Cch 5 μM and 10 μM was evaluated in the following frequency bands: Delta‐Theta (1–15 Hz), Beta (16–28 Hz), Slow Gamma (29–45 Hz), and Fast Gamma (70–200 Hz) for the groups treated with vehicle (black, *n* = 423 recording sites; 9 slices), Aβ (red, *n* = 282 recording sites; 12 slices), cIH (blue, *n* = 235 recording sites; 6 slices) and combination (purple, *n* = 282 recording sites; 6 slices). Circles denote a *p* < 0.05 vs. Basal, * denotes a *p* < 0.05 vs. Veh; two‐way ANOVA followed by *t*‐tests corrected for multiple comparisons by Benjamini and Hochberg's False Discovery Rate.

**FIGURE 4:**
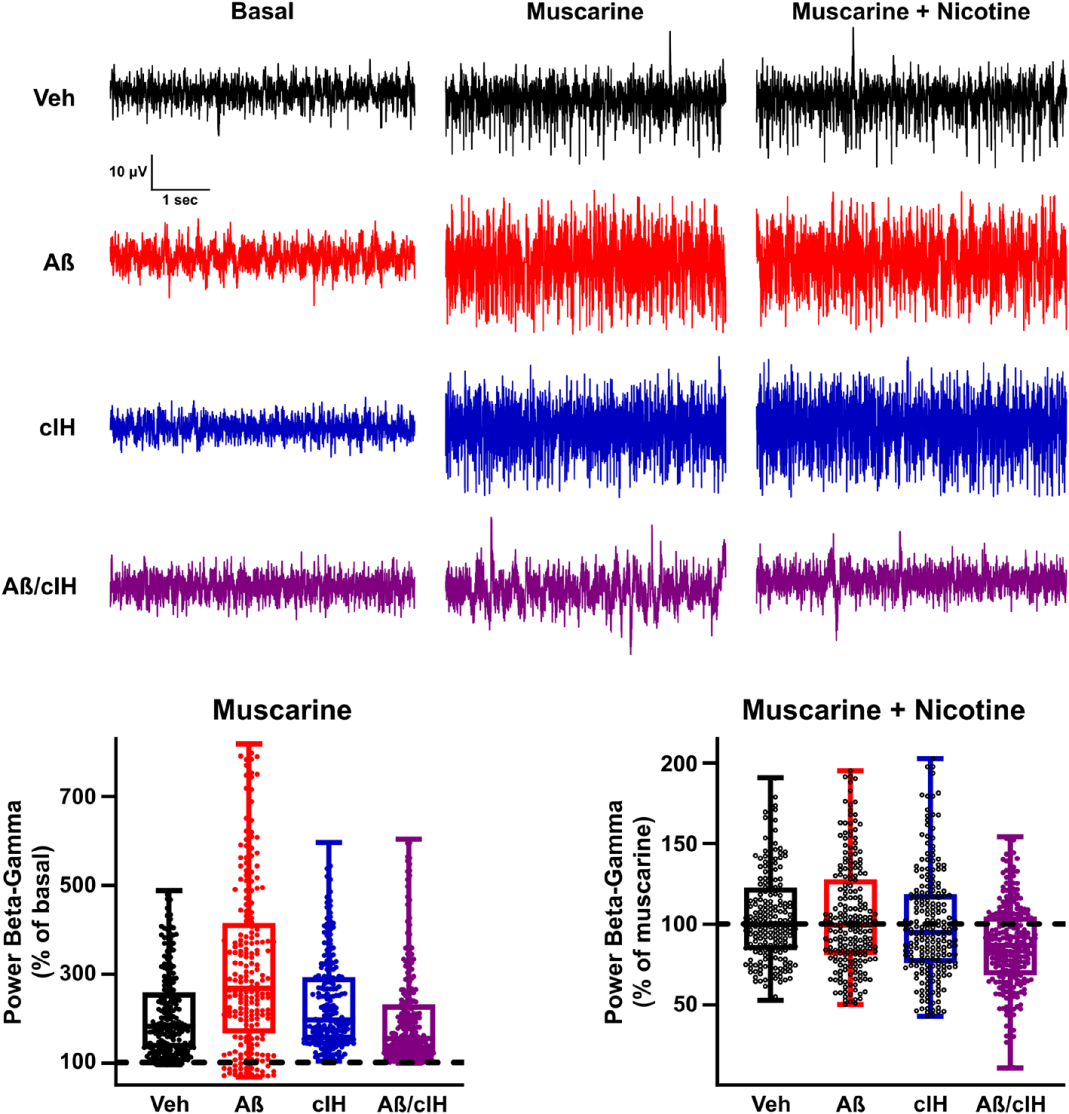
Modulation of hippocampal population activity by muscarine and nicotine. Representative broadband traces of hippocampal recordings before (basal) and during the sequential application of muscarine (after basal) and nicotine (after muscarine) are shown for all the experimental groups. Normalized power (previous condition set as 100%) of the Beta/Gamma activity (16–200 Hz) for the groups treated with vehicle (black, *n* = 212 recording sites; 4 slices), Aβ (red, *n* = 212 recording sites; 4 slices), cIH (blue, *n* = 212 recording sites; 4 slices) and combination (purple, *n* = 265 recording sites; 5 slices) in response to the sequential bath application of muscarine (after basal) and nicotine (after muscarine). Colored circles as the actual group denote a significant difference (*p* < 0.05) vs. previous condition; one‐way ANOVA followed by *t*‐tests corrected for multiple comparisons by Benjamini and Hochberg's False Discovery Rate.

**FIGURE 5:**
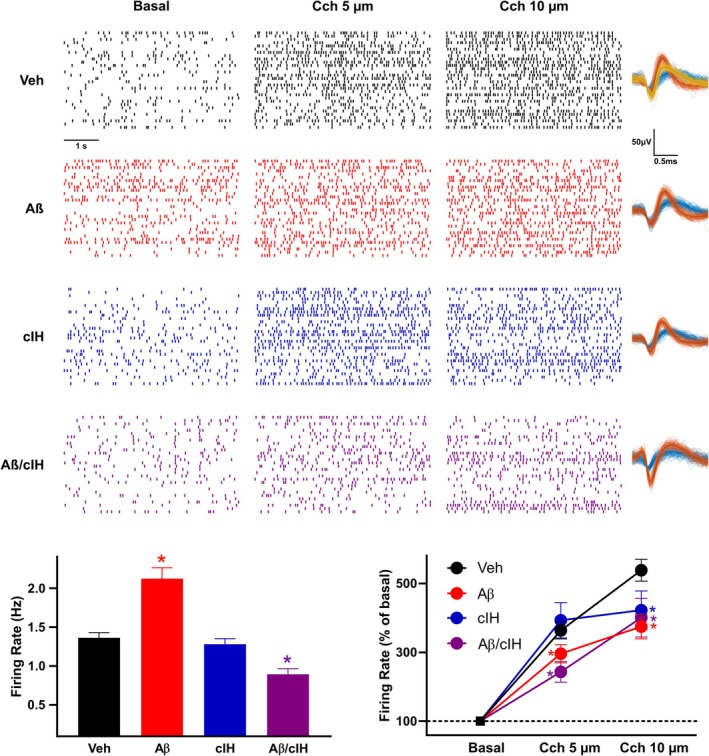
Modulation of neuronal firing by carbachol (Cch). Representative raster plots of hippocampal neuronal firing (one unit per row) before (basal) and during the bath application of Cch 5 μM and 10 μM are shown for all the experimental groups. Examples of spikes clustered to single units are presented for each group. Bottom left graph: Firing frequency for the groups treated with vehicle (black, *n* = 1573 units; 9 slices), Aβ (red, *n* = 812 units; 9 slices), cIH (blue, *n* = 935 units; 6 slices) and their combination (purple, *n* = 754 units; 6 slices). Bottom right graph: Normalized (basal set as 100%) firing response to Cch 5 μM and 10 μM of the same groups. Circles denote a *p* < 0.05 vs. Basal, * denotes a *p* < 0.05 vs. Veh; one‐way ANOVA followed by *t*‐tests corrected for multiple comparisons by Benjamini and Hochberg's False Discovery Rate.

**FIGURE 6:**
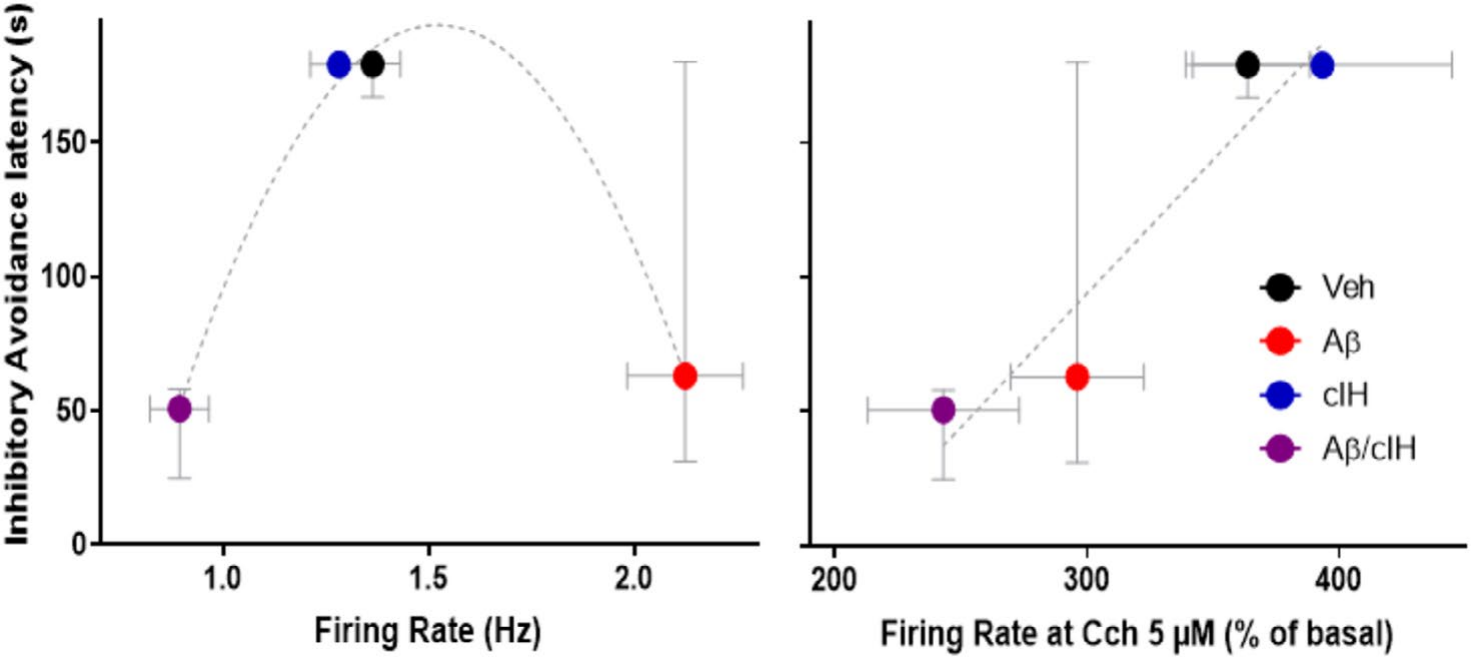
Relationships between firing in basal conditions and its response to carbachol (Cch) with aversive memory. Left graph: Relationship between basal firing and performance during the aversive memory test for the groups treated with vehicle (black), Aβ (red), cIH (blue) and combination (purple). The points can be adjusted to a centered second order quadratic function (dotted line; *R* squared = 0.99). Right graph: Relationship between firing to Cch 10 μM and performance during the aversive memory test for all the experimental groups. The points can be adjusted trough a linear regression (dotted line; *R* squared = 0.99); *F*‐statistic.

Differences in the preference index for the NOR and NL tasks were assessed using the non‐parametric Wilcoxon signed‐rank test for paired samples (Figure 1, top‐left and bottom‐left panels). Latency differences in the “inhibitory avoidance training” task were evaluated with a Wilcoxon rank‐sum test for independent samples (Figure 1, top‐right and bottom‐right panels). To analyze differences in ChAT‐positive cells and in ChAT‐positive axon/terminals a Kruskal‐Wallis was performed (Figure 2). To analyze differences in LFP power change induced by Cch application (0, 5, and 10 μM) across experimental groups (Veh, Aβ, cIH, and Aβ + cIH), a two‐way ANOVA was performed, followed by post hoc t‐tests corrected for multiple comparisons using Benjamini and Hochberg's False Discovery Rate (FDR; Figure 3). To analyze differences in LFP power change induced by muscarine and then by nicotine applications across experimental groups (Veh, Aβ, cIH, and Aβ + cIH), a one‐way ANOVA was performed followed by post hoc t‐tests and FDR (Figure 4). Basal firing rate differences among experimental groups (Figure 5, upper‐left panel) were examined using a one‐way ANOVA followed by post hoc *t*‐tests and FDR, while normalized firing rate differences in response to Cch application (Figure 5, lower‐left panel) were analyzed with a two‐way ANOVA followed by post hoc t‐tests and FDR. The relationship between basal firing rates (Hz) and escape latencies was analyzed using a quadratic function (Figure 6, left panel). The relationship between normalized firing rates in response to Cch 5 μM (% of basal) and escape latencies was analyzed using linear regression (Figure 6, right panel), and the statistical significance of the model was assessed with the *F*‐statistic. To analyze differences in microglial morphology a Kruskal‐Wallis followed by Dunn test was performed (Figure S1). To analyze differences in microglial number a one‐way ANOVA was performed (Figure S1). To analyze differences in Aβ staining one‐way ANOVA followed by Dunnett tests vs. vehicle was performed (Figure S2). Statistical significance was set at an alpha (α) level of 0.05. Statistical analyses were performed in Matlab (Version 2020a).

## 3 Results

### 3.1 Aβ and cIH Slightly Affect Mice Memory, Which Is Exacerbated by Their Combination

Mice that similarly explored two objects when first encountered during familiarization were included (85% of them; Xolalpa‐Cueva et al. 2022). After familiarization, control animals spent more time exploring the novel object (NOR *P*_idx_ = 67.62; *p* < 0.01) and the object displaced to a novel location (NL *P*_idx_ = 57.54; *p* < 0.01; Figure 1). The Aβ, cIH, and Aβ + cIH groups exhibited normal NOR (*P*_idx_ = 68.45, 65.51 and 66.85; respectively; *p* < 0.05). In contrast, all three groups failed to detect the object displaced to a novel location (*P*_idx_ = 50.96, 52.48 and 51.05, respectively; *p* > 0.1; Figure 1).

In the inhibitory avoidance test, all animals exhibited a similarly short training latency but exhibited differences 1 day after the foot‐shock (Figure 1). All but two control animals (2/9) avoided the aversive chamber during the 180 s‐long memory evaluation (median latency of 179.09 s). In contrast, half of the animals administered with Aβ (8/15) visited the aversive chamber during memory evaluation (group median latency = 62.93 s, *p* = 0.056). All but two animals subjected to cIH (2/10) avoided the aversive chamber during memory evaluation, and their group median latency (178.96 s) was not different from control animals (*p* = 0.93). All animals that received the combination of Aβ and cIH visited the aversive chamber during memory evaluation, and their median latency (50.66 s) was significantly shorter than control animals (*p* < 0.01).

### 3.2 Aβ, cIH or Their Combination Do Not Damage Septal Cholinergic Neurons or Cholinergic Hippocampal Innervation

Evaluation of ChAT‐positive cells and axon/terminals in the septum and hippocampus, respectively, revealed that Aβ, cIH, or their combination do not affect them (Figure 2). Namely, control animals exhibited 21.95 ± 3.22 ChAT‐positive cells in an area of 6 × 10^5^ μm^2^ located in the septum, which is similar to the number of ChAT‐positive cells found in the same area of animals treated with Aβ, cIH, or Aβ + cIH (25.13 ± 3.40, 27.53 ± 3.61 and 17 ± 3.01 ChAT‐positive cells; respectively; *p* > 0.05; Figure 2). On the other hand, in control animals, 11.35% ± 1.09% of the area at the interface between the subiculum and CA1 hippocampal regions is covered by ChAT‐positive axon/terminals, which is similar to the area covered by these same terminals in animals treated with Aβ, cIH, or Aβ + cIH (11.67% ± 1.27%, 11.77% ± 0.83% and 9.02% ± 0.86%; respectively; *p* > 0.05; Figure 2).

### 3.3 Combination of Aβ and cIH Strongly Disrupts Hippocampal Network Cholinergic Modulation

Evaluation of the LFP in slices obtained from control animals revealed that bath application of Cch (Figure 3) significantly decreases slow activity (*δ*/*θ*, 1–15 Hz) at the high concentration tested (10 μM; 66.89% ± 3.95% of basal; *p* < 0.01), while increasing fast network activity (*β*, 16–28 Hz; *sγ*, 29–45 Hz; and *fγ*, 70–200 Hz) at both tested concentrations (5 and 10 μM *p* < 0.01). The magnitude of these increments was directly related to their band frequencies (i.e., *β* = 151.88% ± 6.05% of basal; *sγ* = 172.83% ± 7.16% of basal and *fγ* = 216.04% ± 11.19% of basal at Cch 10 μM).

In slices from animals administered with Aβ, the Cch‐induced reduction of *δ*/*θ* activity was absent at Cch 5 μM (*p* = 0.68) and only a trend was observed at 10 μM (*p* = 0.062). Despite that, in these slices, Cch increased *β*, *sγ*, and *fγ* activity (Figure 3); the increase at Cch 5 μM was smaller than control slices (*p* < 0.05). In slices from animals subjected to cIH, the Cch‐induced reduction in *δ*/*θ* activity was higher than the one hinted in control slices at Cch 5 μM (58.19% ± 5.27% of basal; *p* < 0.01). In these slices, Cch increased *β* activity as in control slices (*p* > 0.1). In contrast, the Cch‐induced increases of *sγ* and *fγ* activity were smaller than control slices (*p* < 0.05). In slices obtained from animals subjected to cIH and Aβ, Cch induced an exacerbated reduction in *δ*/*θ* activity (*p* < 0.01). In these slices, Cch 5 μM only tended to increase *β* activity (*p* = 0.083) and even reduced it at 10 μM (*p* < 0.05). In these slices, Cch failed to modulate *sγ* activity (*p* > 0.1) and the Cch‐induced increase in *fγ* activity was smaller than control slices (*p* < 0.05).

Evaluation of the *β*/*γ* activity in slices obtained from control animals revealed that bath application of muscarine 2.5 μM (Figure 4) significantly increases this fast activity (median power of 209.79% of basal; *p* < 0.01), which is also observed in slices obtained from animals treated with Aβ (median power of 315.05% of basal; *p* < 0.01) or cIH (median power of 248.83% of basal; *p* < 0.01). However, this increase was significantly smaller in slices obtained from animals treated with the combination of Aβ + cIH (median power of 199.78% of basal; *p* < 0.05). Subsequent nicotine 100 μM administration did not significantly affect *β*/*γ* activity in slices obtained from control animals, or from animals treated with either Aβ or with cIH (median power of 105.39, 104.48 and 99.79% of basal, respectively; *p* > 0.05; Figure 4). However, nicotine 100 μM significantly reduced *β*/*γ* activity in slices obtained from animals treated with the combination of Aβ and cIH (median power of 86.97% of basal; *p* < 0.01).

### 3.4 Combination of Aβ and cIH Strongly Disrupts Hippocampal Firing, Cholinergic Modulation, and Its Relationship with Aversive Memory

Evaluation of hippocampal neuronal firing and its modulation by Cch (Figure 5) reveals that control animals exhibited a firing frequency of 1.36 ± 0.07 Hz, which was significantly increased upon bath application of Cch (5 μM = 363.31% ± 24.58% and 10 μM = 538.59% ± 31.62%; *p* < 0.01). Neurons from the Aβ group exhibited a higher firing frequency than control slices (2.12 ± 0.14 Hz; *p* < 0.01). In these slices, the Cch‐induced increase in firing frequency was smaller than control slices at 5 μM (296.04% ± 26.44%; *p* < 0.05) and 10 μM (375.00% ± 35.57%; *p* < 0.01). In slices from the cIH group, neurons exhibited a normal firing frequency (1.28 ± 0.07; *p* > 0.05). In these slices, the Cch‐induced increase in firing frequency was reduced when tested at 10 μM (422.36% ± 55.88%; *p* < 0.05). In slices from the cIH + Aβ group, neurons exhibited a reduced firing frequency (0.83 ± 0.07 Hz; *p* < 0.01) and a reduced responsiveness to Cch 5 μM (243.07% ± 0.30%) and 10 μM (400.89% ± 56.20%; *p* < 0.01).

Reduced aversive memory recall was related to either an increase in basal firing frequency in the group treated with Aβ or with a decrease in basal firing frequency in the group treated with the combination of Aβ and cIH (Figure 6). In fact, the relationship between memory performance in the inhibitory avoidance test and basal firing frequency for all groups was very well fitted to a centered second order quadratic function (*R*‐squared = 0.99; Figure 6). Alternatively, the reduced responsiveness to Cch 5 μM in the Aβ and the Aβ + cIH groups was correlated with poorer performance in the aversive memory test (Figure 6). Moreover, the relationship between memory performance in the inhibitory avoidance test and the change in firing frequency in the presence of Cch 5 μM was very well described by a linear function (*R*‐squared = 0.99; *p* < 0.05; Figure 6).

## 4 Discussion

In this study, we found that animals subjected to cIH or administered Aβ have a deficit in NL but spared NOR. Object recognition resisted even in animals treated with the combination of cIH and Aβ. In contrast, this combination produced an additive amnesic effect in the inhibitory avoidance test. We also found that cIH and Aβ induce similar alterations (with minimal divergences) in the modulation of hippocampal network activity induced by different cholinomimetics but, when combined, dramatically depress Cch‐induced hippocampal network excitation and even lead to Cch‐induced hippocampal network inhibition in response to a high concentration of Cch. The latter coincides with the inhibition of the hippocampal network induced by nicotine only in those animals treated with the combination of cIH and Aβ. When evaluated individually, cIH and Aβ produce variable changes in the basal firing rate and/or its response to Cch, but when combined, they induce a generalized reduction in neuronal firing rate and its responsiveness to Cch. When correlating neuronal activity with behavior, we found that the reduced performance in the inhibitory avoidance test correlated with both hyperexcitable neurons in animals administered Aβ and with hypoexcitable neurons in animals exposed to the combination of Aβ and cIH. Moreover, poor memory in this test also correlated with a reduced responsiveness to Cch.

NL evaluates spatial recognition memory and is highly dependent on hippocampal function (Winters et al. 2008; Oliveira‐Lima et al. 2024; Barker and Warburton 2011; Mendez et al. 2015). In contrast, NOR evaluates non‐spatial recognition memory and depends on a more distributed circuitry, including the perirhinal cortex (Winters et al. 2008; Barker and Warburton 2011; Mendez et al. 2015). Thus, there is a strong neuronal recruitment of CA1/subiculum during NL (but not of perirhinal neurons), whereas there is a strong neuronal recruitment in the perirhinal cortex during NOR (but not of hippocampal neurons; Mendez et al. 2015). As observed in our results and previous reports, in pathological conditions, spatial recognition memory is more sensitive to disruption than non‐spatial object recognition when measured in identical conditions (Davis et al. 2013; Jablonski et al. 2013; Creighton et al. 2019; Olave et al. 2022; Jiao et al. 2022; Wang et al. 2023). This exact scenario (deficits in NL but spared NOR in identical pathological conditions) has also been observed when Aβ is elevated (Davis et al. 2013; Creighton et al. 2019; however, see Oliveira‐Lima et al. 2024). Moreover, even when both types of memory are simultaneously affected (Ehret et al. 2023; Oliveira‐Lima et al. 2024), NOR is easier to recover after neuroprotection (Pitsikas and Tarantilis 2017; Ehret et al. 2023), indicating that spatial recognition memory is more susceptible than non‐spatial recognition memory (Jablonski et al. 2013), presumably because NL is based on less redundant information and depends on a less distributed neural circuitry (Winters et al. 2008; Barker and Warburton 2011; Mendez et al. 2015; Pitsikas and Tarantilis 2017).

Considering that Aβ is accumulated and contributes to both AD and OSA pathophysiology (Bubu et al. 2020; Peña‐Ortega 2013, 2019; Ju et al. 2019; Owen et al. 2021; Kong et al. 2021; Bu et al. 2015; Sharma et al. 2018; Przybylska‐Kuć et al. 2019; Bhuniya et al. 2022; Lao et al. 2022), that OSA treatment can reduce the cognitive impairment in AD patients (Bubu et al. 2020), and that cIH induces Aβ overproduction in rodents (Li et al. 2009; Ng et al. 2010; Shiota et al. 2013; Kazim et al. 2022), we hypothesize that Aβ contributes to cIH‐induced impairment in the NL (Davis et al. 2013; Salgado‐Puga et al. 2017; Creighton et al. 2019), which could also be explained by the inhibition of neuronal network activity by cIH and Aβ (Salgado‐Puga et al. 2017; Hernández‐Soto et al. 2019). Moreover, their additive amnesic effects in the aversive memory, which coincide with the evidence that cIH exacerbates cognitive decline in AD transgenic mice (Shiota et al. 2013; Kazim et al. 2022), could be explained by Aβ overaccumulation (the exogenously applied plus the accumulated by cIH; Ng et al. 2010; Davis et al. 2013; Salgado‐Puga et al. 2017; Creighton et al. 2019; Ehret et al. 2023; Oliveira‐Lima et al. 2024), because it is well known that Aβ induces cognitive decline in a dose‐dependent manner (Puzzo et al. 2012; Salgado‐Puga et al. 2015, 2017). However, despite using three different staining methods (Figure S2), we were not able to detect a consistent increase in Aβ levels induced by Aβ injection, cIH, or their combination (despite that the three types of staining used were able to detect increased labeling in Aβ overproducing transgenic mice; Figure S2). We do not discard that Aβ is increasing in our experimental conditions, but we lacked proper sensitivity to reveal such accumulation.

Several pathological events beyond Aβ accumulation could converge to induce the potentiation of cognitive decline induced when cIH and Aβ are combined, including additive alterations in intracellular transduction pathways (Marciante et al. 2022), additive oxidative stress and/or additive inflammation (Ng et al. 2010; Arias‐Cavieres et al. 2020). Regarding alterations of intracellular transduction pathways, we have previously shown that Aβ‐induced deleterious effects involve the activation of glycogen synthase kinase 3β (GSK‐3β) and some of its targets (Peña‐Ortega et al. 2012; Isla et al. 2016; Salgado‐Puga et al. 2017; Cornejo‐Montes‐de‐Oca et al. 2018), which are also recruited during cIH‐induced pathology (Marciante et al. 2022). Coincidentally, the activation of GSK‐3β and some of its targets is involved in the potentiation of cognitive impairment induced by the combination of cerebral ischemia and Aβ (Song et al. 2013). Regarding oxidative stress and inflammation (Ng et al. 2010; Arias‐Cavieres et al. 2020), there is evidence that cIH‐induced Aβ accumulation can be prevented by the antioxidant melatonin (Ng et al. 2010), which coincides with the finding that cIH‐induced cognitive impairment can be prevented by antioxidant treatment (Arias‐Cavieres et al. 2020). In addition, the potentiation of memory impairment induced by Aβ and sleep disruption, which is related to the presence of neuroinflammation (Qian et al. 2022), can be prevented with the TNF‐α neutralizing monoclonal antibody infliximab (Kincheski et al. 2017). Thus, the amnesic effects observed after the combination of Aβ and cIH can also be due to the synergistic actions of Aβ and several pro‐inflammatory cytokines (LaRocca et al. 2021). Of note, neuroinflammation, cIH, and Aβ can alter neural network function in a similar, and perhaps convergent, fashion (Lee et al. 2012; Peña‐Ortega 2013, 2019; D'Rozario et al. 2017; Xiromeritis et al. 2011; Appleton et al. 2019; Muñoz‐Torres et al. 2020; Liu et al. 2021; Villasana‐Salazar et al. 2020; Hernández‐Soto et al. 2021; Kang et al. 2021).

As already mentioned, AD and OSA patients exhibit a similar EEG slowing (Muñoz‐Torres et al. 2020) that can be partially corrected with OSA treatment (Liguori et al. 2017), even in AD patients (Owen et al. 2021). A component of the EEG slowing is a reduction in fast oscillations, as shown in this study, that has been associated with alterations in synaptic and intrinsic properties (Salgado‐Puga et al. 2017; Arias‐Cavieres et al. 2020; Gutiérrez‐Lerma et al. 2013; Torres‐Flores and Peña‐Ortega 2022), alterations in the integrity of populations of interneurons (Merchant et al. 2012; Rubin et al. 2020) or in the cholinergic modulation of neuronal network properties (Gutiérrez‐Lerma et al. 2013; Qian et al. 2022). Here we also found that the reduction in neuronal cholinergic modulation in the presence of Aβ, and its combination with cIH, is related to mnemonic impairment. As for the cognitive decline discussed in the previous paragraph (Torres‐Flores and Peña‐Ortega 2022), these alterations can emerge from a complex combination of pathological processes associated with alterations in intracellular transduction pathways (Pena‐Ortega et al. 2012; Isla et al. 2016; Salgado‐Puga et al. 2017; Cornejo‐Montes‐de‐Oca et al. 2018; Marciante et al. 2022), oxidative stress and inflammation (Ng et al. 2010; Arias‐Cavieres et al. 2020; Qian et al. 2022), disturbances in the cholinergic system itself (Gutiérrez‐Lerma et al. 2013; Qian et al. 2022), changes in synaptic connectivity (Salgado‐Puga et al. 2017; Arias‐Cavieres et al. 2020; Torres‐Flores and Peña‐Ortega 2022) and/or alterations in ion channels (Peña et al. 2006; Torres‐Flores and Peña‐Ortega 2022).

Activation of both nicotinic and muscarinic receptors exerts a complex combination of inhibitory and excitatory actions on hippocampal network activity (Pan and Williams 1994; Gulledge and Stuart 2005; Gulledge and Kawaguchi 2007; Widmer et al. 2006; McQuiston and Madison 1999; Somogyi and de Groat 1992; Bell et al. 2013; Wang et al. 2015; Yang et al. 2020), that depends on the recruited transduction pathway(s) (Jope et al. 1997; Shiozaki and Iseki 2004; Tsang et al. 2006; Janíčková et al. 2013), on their diverse actions on pyramidal cells and interneurons (McQuiston and Madison 1999; Widmer et al. 2006; Dannenberg et al. 2017; Yang et al. 2020), as well as on the network state (Somogyi and de Groat 1992; Bell et al. 2013; Plata et al. 2013; Wang et al. 2015; Dannenberg et al. 2017; Bueno‐Junior et al. 2017; Yang et al. 2020). We found that the combination of Aβ and cIH reduces the excitatory modulation induced by muscarine, which can be associated with a reduction in the expression of type 1, 3, and 5 muscarinic receptors (Mash et al. 1985; Reinikainen et al. 1987; Wang et al. 1992; Flynn et al. 1995; Rodríguez‐Puertas et al. 1997; Hambrecht et al. 2007), or their coupling with their intracellular pathways (Jope et al. 1997; Shiozaki and Iseki 2004; Tsang et al. 2006; Janíčková et al. 2013). We also found that in slices obtained from animals treated with the combination of Aβ and cIH, nicotine inhibits hippocampal activity (Figure 4). This inhibitory nicotinic modulation has been previously reported beyond the hippocampus (de la Garza et al. 1987; Wong and Gallagher 1989, 1991; Mansvelder et al. 2006; Plata et al. 2013; Sigalas et al. 2015; Wang et al. 2015; Bueno‐Junior et al. 2017) and could be pathologically revealed in this circuit when Aβ and cIH are combined, contributing to the additive inhibitory actions on hippocampal activity.

Regarding the effects of cIH and Aβ on hippocampal neuronal firing, we found that Aβ increases basal firing, while its combination with cIH reduces it (Figure 5). The induction of hyperexcitability by Aβ has been extensively reported by several groups, including ours (Eslamizade et al. 2015; Palop and Mucke 2016; Alcantara‐Gonzalez et al. 2019; Torres‐Flores and Peña‐Ortega 2022; Robles‐Gómez et al. 2023) and has been closely related to the degree of cognitive impairment (Palop and Mucke 2016; Torres‐Flores and Peña‐Ortega 2022; Robles‐Gómez et al. 2023). In contrast, little is known about the reduction in firing induced by the combination of Aβ and cIH. This hypoactivity could be induced by changes in synaptic connectivity (Hasselmo et al. 1992; Balleza‐Tapia et al. 2010; Salgado‐Puga et al. 2017; Flores‐Martínez and Peña‐Ortega 2017; Khuu et al. 2019; Alcantara‐Gonzalez et al. 2019; Arias‐Cavieres et al. 2020; Martínez‐García et al. 2021; Torres‐Flores and Peña‐Ortega 2022) and/or the properties of ion channels (Peña et al. 2006; Torres‐Flores and Peña‐Ortega 2022) that not just reduce basal firing activity but additionally impair the firing rate increase in response to cholinergic modulation (Figure 5). There is plenty of evidence that a hypoactive state, as the one induced by the combination of cIH and Aβ, is closely related to profound cognitive impairments (Zarrindast et al. 2002; Peña‐Ortega 2013, 2019) and could be the foundation for the reduction in avoidance behavior (Zarrindast et al. 2002). As previously shown (Zarrindast et al. 2002; Robles‐Gómez et al. 2023), here we found that both a hyperactive and a hypoactive hippocampus can be related to memory impairment (Figure 6), indicating that diverting neurons from their “homeostatic” functional range is enough to alter neuronal processing and memory (Gill et al. 2000; Zarrindast et al. 2002; Palop and Mucke 2016; Robles‐Gómez et al. 2023).

We found that Aβ alone or in combination with cIH reduces the increase in neuronal firing induced by a low concentration of Cch (Figure 5). Evaluating firing activity in the presence of Cch in vitro is a useful strategy that simulates the well‐known increase in acetylcholine during “online” hippocampus‐dependent memory processes (Hasselmo et al. 1992; Rodríguez‐García and Miranda 2016) and evokes distinct hippocampal network states by increasing concentrations of Cch (Fellous and Sejnowski 2000). As already discussed, the reduction in firing response in the presence of Cch could be related to disturbances in cholinergic transmission (Hasselmo et al. 1992; Hambrecht et al. 2007; Gutiérrez‐Lerma et al. 2013; Tang et al. 2020; Qian et al. 2022), in cholinergic receptors (Mash et al. 1985; Reinikainen et al. 1987; Wang et al. 1992; Flynn et al. 1995; Rodríguez‐Puertas et al. 1997; Hambrecht et al. 2007) and/or in their coupling with intracellular pathways (Jope et al. 1997; Shiozaki and Iseki 2004; Tsang et al. 2006; Janíčková et al. 2013), which could be the basis for memory deterioration (Hasselmo et al. 1992; Gill et al. 2000; Sun et al. 2017). In fact, we observed that the reduced responsiveness to Cch in the Aβ and the Aβ + cIH groups correlated with poorer performance in the aversive memory test (Figure 6), which coincides with computational and experimental evidence that cholinergic potentiation of neuronal firing promotes memory processing (Hasselmo et al. 1992; Gill et al. 2000; Sun et al. 2017).

Epidemiological, clinical, and experimental evidence has supported an interdependent relationship between OSA and AD, but few studies have explored the cellular and network mechanisms behind this association. Here we showed that cIH or Aβ can produce convergent, but also slightly different effects on cognition and hippocampal network function, revealing a potential neural substrate for OSA and AD pathophysiologies and their co‐morbidity. The identification of the specific cellular processes responsible for these interactions could help treat patients suffering either one, or the two conditions, but also could help to prevent the presence of this comorbidity in patients enduring either one of them.

## Conflicts of Interest

The authors declare no conflicts of interest.

## Supporting information


**Figure S1.** Microglial density and morphology after intracerebroventricular injection of 5 μL of vehicle. Representative micrographs of hippocampal Iba1 + microglia from the administered hemisphere (injected) and its contralateral side, along with microglia from sham animals (left). A representative microglial cell is magnified for each micrograph and binarized on the right. Graphs show the quantification of microglial density and several morphometric parameters of microglia from the described sites. Filled circles denote a *p* < 0.05 vs. both contralateral and sham microglia, whereas half‐filled circles denote a significant difference (*p* < 0.05) only vs. contralateral microglia. Kruskal‐Wallis followed by Dunn test.
**Figure S2.** Amyloid‐beta (Aβ) labeling. Representative micrographs of the β42 antibody immunostaining, as well as histological labeling with Thioflavin S and Thiazine Red for two Aβ over‐producing transgenic mice (Tg5x and Tg3x) as well as for the animals treated with vehicle (Veh), with Aβ, chronic intermittent hypoxia (cIH) and their combination with Aβ (Aβ/cIH). For the transgenic animals, a close‐up of the amyloid beta aggregates, exemplified with the arrowheads, was included. No amyloid beta accumulation was observed in any of the other experimental groups. Graphs display the fluorescence intensity for each labeling. The mean and standard errors are included with the following color code: vehicle in black (*n* = 12 sections, 4 animals), Aβ in red (*n* = 12 sections, 4 animals), cIH in blue (*n* = 9 sections, 3 animals), and combination in purple (*n* = 12–15 sections, 4–5 animals). Aβ over‐producing transgenic mice are also represented in red (*n* = 3 sections, 1 animal). Colored circles as the actual group denote a significant difference (*p* < 0.05) vs. Veh. Kruskal‐Wallis followed by Dunnett test vs. Veh.

## Data Availability

The data that support the findings of this study are available from the corresponding author upon reasonable request.
