# Supplementary material for: Combined Effects of Intermittent Hypoxia and Amyloid Beta on Hippocampal Activity, Its Cholinergic Modulation, and Memory
Source: Hippocampus. 2025 Jun 11;35(4):e70017. doi: 10.1002/hipo.70017 (PMC12152712; doi:10.1002/hipo.70017)
Supplement: Supplementary file 1 — Figure S1. Microglial density and morphology after intracerebroventricular injection of 5 μL of vehicle. Representative micrographs of hippocampal Iba1 + microglia from the administered hemisphere (injected) and its contralateral side, along with microglia from sham animals (left). A representative microglial cell is magnified for each micrograph and binarized on the right. Graphs show the quantification of microglial density and several morphometric parameters of microglia from the described sites. Filled circles denote a p < 0.05 vs. both contralateral and sham microglia, whereas half‐filled circles denote a significant difference (p < 0.05) only vs. contralateral microglia. Kruskal‐Wallis followed by Dunn test. Figure S2. Amyloid‐beta (Aβ) labeling. Representative micrographs of the β42 antibody immunostaining, as well as histological labeling with Thioflavin S and Thiazine Red for two Aβ over‐producing transgenic mice (Tg5x and Tg3x) as well as for the animals treated with vehicle (Veh), with Aβ, chronic intermittent hypoxia (cIH) and their combination with Aβ (Aβ/cIH). For the transgenic animals, a close‐up of the amyloid beta aggregates, exemplified with the arrowheads, was included. No amyloid beta accumulation was observed in any of the other experimental groups. Graphs display the fluorescence intensity for each labeling. The mean and standard errors are included with the following color code: vehicle in black (n = 12 sections, 4 animals), Aβ in red (n = 12 sections, 4 animals), cIH in blue (n = 9 sections, 3 animals), and combination in purple (n = 12–15 sections, 4–5 animals). Aβ over‐producing transgenic mice are also represented in red (n = 3 sections, 1 animal). Colored circles as the actual group denote a significant difference (p < 0.05) vs. Veh. Kruskal‐Wallis followed by Dunnett test vs. Veh. [file HIPO-35-0-s001.docx]

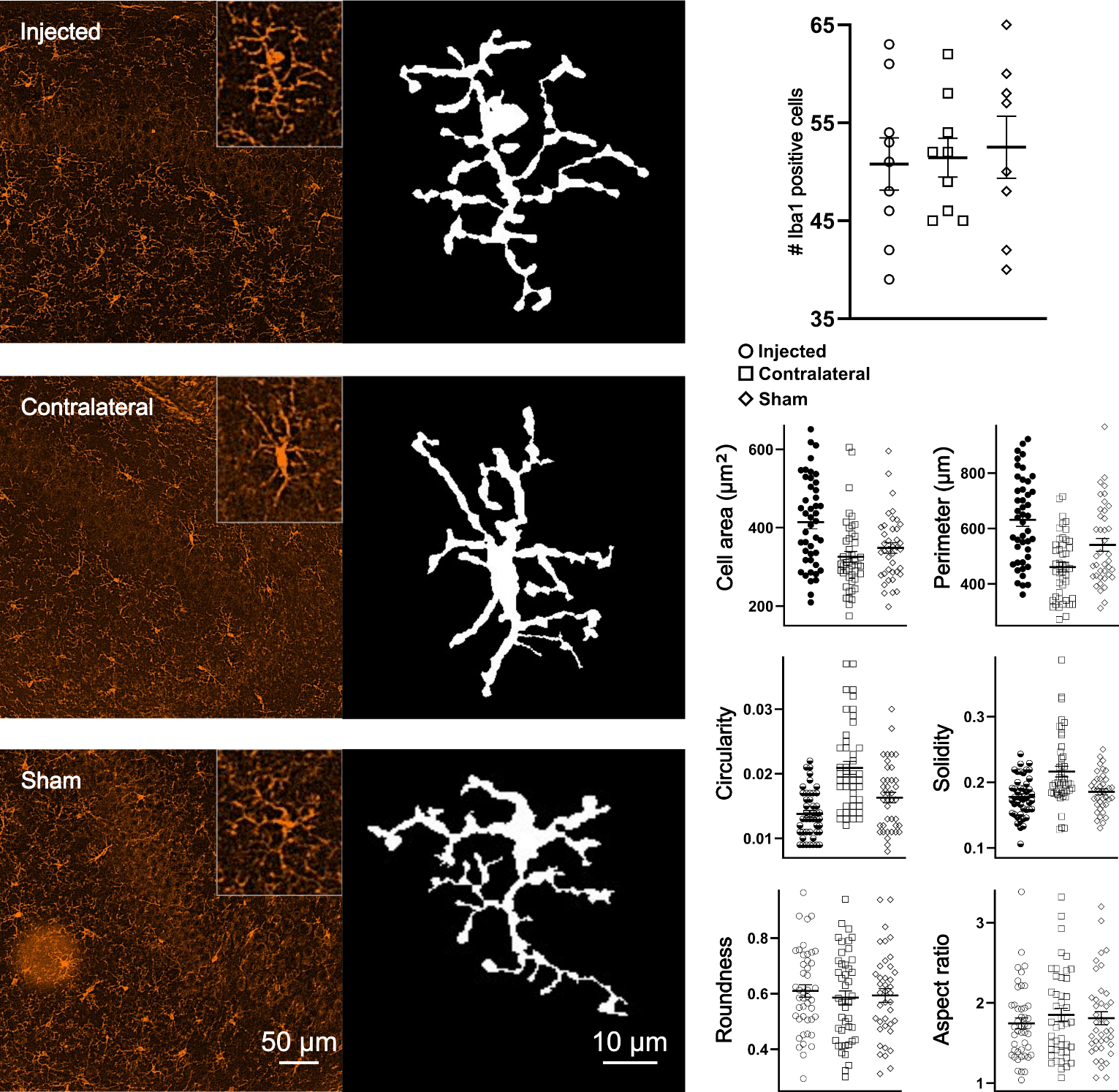


**Supplementary Figure 1.-** Microglial density and morphology after intracerebroventricular injection of 5 µl of vehicle. Representative micrographs of hippocampal Iba1+ microglia from the administered hemisphere (injected) and its contralateral side, along with microglia from sham animals (left). A representative microglial cell is magnified for each micrograph and binarized on the right. Graphs show the quantification of microglial density and several morphometric parameters of microglia from the described sites. Filled circles denote a p < 0.05 vs both contralateral and sham microglia, whereas half-filled circles denote a p < 0.05 only vs contralateral microglia. Kruskal-Wallis followed by Dunn test.


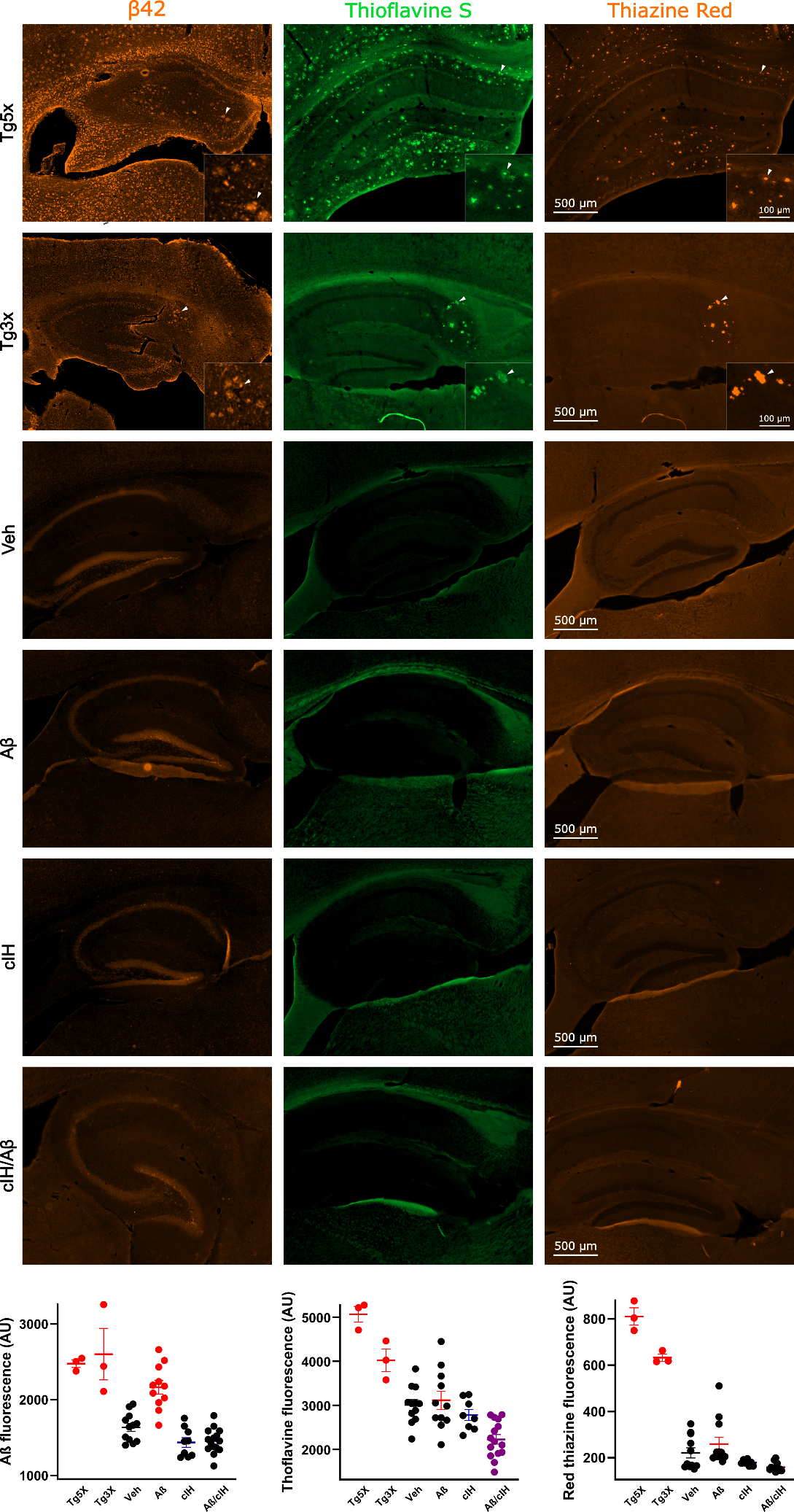


**Supplementary Figure 2.-** Amyloid-beta (Aβ) labeling. Representative micrographs of the β42 antibody immunostaining, as well as histological labeling with Thioflavin S and Thiazine Red for two Aβ over-producing transgenic mice (Tg5x and Tg3x) as well as for the animals treated with vehicle (Veh), with Aβ, chronic intermittent hypoxia (cIH) and their combination with Aβ (Aβ/cIH). For the transgenic animals, a close-up of the amyloid beta aggregates, exemplified with the arrowheads, was included. No amyloid beta accumulation was observed in any of the other experimental groups. Graphs display the fluorescence intensity for each labeling. The mean and standard errors are included with the following color code: vehicle in black (n=12 sections, 4 animals), Aβ in red (n=12 sections, 4 animals), cIH in blue (n=9 sections, 3 animals), and combination in purple (n=12-15 sections, 4-5 animals). Aβ over-producing transgenic mice are also represented in red (n=3 sections, 1 animal). Colored circles as the actual group denote a p < 0.05 vs Veh. Kruskal-Wallis followed by Dunnett test vs Veh.
